# A novel trifunctional IgG-like bispecific antibody to inhibit HIV-1 infection and enhance lysis of HIV by targeting activation of complement

**DOI:** 10.1186/1743-422X-7-142

**Published:** 2010-06-29

**Authors:** Leili Jia, Yuanyong Xu, Chuanfu Zhang, Yong Wang, Huihui Chong, Shaofu Qiu, Ligui Wang, Yanwei Zhong, Weijing Liu, Yansong Sun, Fei Qiao, Stephen Tomlinson, Hongbin Song, Yusen Zhou, Yuxian He

**Affiliations:** 1 Institute of Disease Control and Prevention, Academy of Military Medical Science, Beijing 100071, PR China; 2 Institute of Pathogen Biology, Chinese Academy of Medical Sciences and Peking Union Medical College, Beijing 100730, PR China; 3 Department of Microbiology and Immunology, Medical University of South Carolina, Charleston, South Carolina 29425, USA; 4 State Key Laboratory of Pathogen and Biosecurity, Beijing Institute of Microbiology and Epidemiology, Beijing 100071, PR China; 5 The 302nd Hospital of People's Liberation Army, Beijing 100039, PR China

## Abstract

**Background:**

The complement system is not only a key component of innate immunity but also provides a first line of defense against invading pathogens, especially for viral pathogens. Human immunodeficiency virus (HIV), however, possesses several mechanisms to evade complement-mediated lysis (CoML) and exploit the complement system to enhance viral infectivity. Responsible for this intrinsic resistance against complement-mediated virolysis are complement regulatory membrane proteins derived from the host cell that inherently downregulates complement activation at several stages of the cascade. In addition, HIV is protected from complement-mediated lysis by binding soluble factor H (fH) through the viral envelope proteins, gp120 and gp41. Whereas inhibition of complement activity is the desired outcome in the vast majority of therapeutic approaches, there is a broader potential for complement-mediated inhibition of HIV by complement local stimulation.

**Presentation of the hypothesis:**

Our previous studies have proven that the complement-mediated antibody-dependent enhancement of HIV infection is mediated by the association of complement receptor type 2 bound to the C3 fragment and deposited on the surface of HIV virions. Thus, we hypothesize that another new activator of complement, consisting of two dsFv (against gp120 and against C3d respectively) linked to a complement-activating human IgG1 Fc domain ((anti-gp120 × anti-C3d)-Fc), can not only target and amplify complement activation on HIV virions for enhancing the efficiency of HIV lysis, but also reduce the infectivity of HIV through blocking the gp120 and C3d on the surface of HIV.

**Testing the hypothesis:**

Our hypothesis was tested using cell-free HIV-1 virions cultivated *in vitro *and assessment of virus opsonization was performed by incubating appropriate dilutions of virus with medium containing normal human serum and purified (anti-gp120 × anti-C3d)-Fc proteins. As a control group, viruses were incubated with normal human serum under the same conditions. Virus neutralization assays were used to estimate the degree of (anti-gp120 × anti-C3d)-Fc lysis of HIV compared to untreated virus.

**Implications of the hypothesis:**

The targeted complement activator, (anti-gp120 × anti-C3d)-Fc, can be used as a novel approach to HIV therapy by abrogating the complement-enhanced HIV infection of cells.

## Background

The human immunodeficiency virus (HIV) causes severe immune deficiency in humans and over 7,000 people are infected everyday [1]. The key to resistance to HIV infection and disease progression resides within the host immune system that consists of two major defense pathways: innate and adaptive immunity [2]. There is a growing recognition that the complement system contributes to HIV replication and pathogenesis [3,4]. In fact, as a first line of defense against pathogenic microorganisms and a mediator between the innate and adaptive immune responses, the complement system is a particular focus of these immune-evasion strategies [4-6]. In human plasma, HIV immediately activates the complement system, even in the absence of HIV-specific antibodies [7]. After seroconversion, the presence of HIV-specific antibodies triggers further activation of the classical complement pathway [11]. Complement activation would be harmful to the virus if the reactions were allowed to go to completion, since their final outcome would be virolysis. HIV, however, has evolved several mechanisms to evade complement-mediated lysis (CoML) and exploit the complement system to enhance viral infectivity [8]. This may be critical, as during opsonization, high amounts of C3-fragments are deposited on the surface of HIV. Also, binding of C3-fragments to gp120 reduces the accessibility of the viral envelope protein [9]. Current therapies for HIV infection using highly active antiretroviral therapy (HAART) are not able to completely eliminate virus and complications of these therapies include severe side effects and viral resistance that may establish latent reservoirs of HIV. There remains a need to develop novel treatments for infected individuals who may no longer respond to or who have significant toxicity from antiretroviral therapy and to prevent HIV transmission [10]. To this end, bispecific antibody (BsAb) constructs may be used to target HIV and infected cells for destruction, resulting in greater control and prevention of infection. We previously reported a targeted complement activator [11], CR2-Fc, and the results shown that CR2-Fc can enhance lysis of HIV (data not show). However, the targets of CR2-Fc are C3d and C3dg, which can be distributed widely when complicating other diseases. Thus, it is interesting to know whether target to HIV envelope could improve the anti-virus efficacy of complement. Here, we hypothesize that a bispecific, trifunctional antibody construct incorporating the disulfide-stabilized Fv fragments (dsFv) against gp120, the dsFv against C3d and Fc promotes destruction of HIV type 1 (HIV-1) by complement.

## Presentation of the hypothesis

### Intrinsic resistance of HIV against the complement system

As mentioned above, HIV is resistant to lysis by the complement MAC C5b-9 [12-17]. Responsible for this intrinsic resistance against complement-mediated virolysis are complement regulatory membrane proteins derived from the host cell, which are acquired by HIV during the budding process [18]. Among them are regulators of complement activation (RCAs) such as accelerating factor (DAF, CD55), membrane cofactor protein (MCP, CD46) or CD59, which -- within a species (human serum--human cells) -- down-regulate complement activation at several stages of the cascade [19-21]. In addition, HIV is protected from complement-mediated lysis by binding soluble factor H (fH) through gp120 and gp41 [22-24]. The crucial role of fH for protection of the virus is evident, since incubation of HIV with fH-depleted sera results in up to 80% of complement-dependent virolysis in the presence of HIV-specific antibodies [23]. Similar to fH-depleted sera that promotes CoML, an fH-derived peptide is able to enhance C3 deposition on HIV infected cells and thus to induce virolysis. However, without intervention HIV remains resistant to human serum [25]. This acquired resistance can further promote infection since fH promoter factor I to cleave activated C3 on the viral surface into iC3b and C3d [24]. C3d remains covalently bound to the target [17]. The resultant opsonized virus thus enhances its infectivity toward CR1, CR2 and CR3-positive cells [24]. Due to these protection mechanisms, opsonized HIV accumulates in all complement-enriched compartments of the host, such as blood, lymphatic tissue (LT), brain, mother's milk, seminal fluid or mucosal surfaces [17]. Strikingly, gp120 is also responsible for recruiting the regulators factor CD59 to the surface of the virus. In addition to inhibition of MAC formation by CD59, the degradation of C3b by factor I and factor H reduces amplification. Whereas inhibition of complement activity is the desired outcome in the vast majority of therapeutic approaches, its local stimulation may be beneficial for the lysis of HIV.

### Bispecific antibodies considered "trifunctional"

The hallmark of monoclonal antibodies is their specificity for a particular antigen that enables them to bind their target precisely *in vivo *while ignoring antigen-negative sites. Monoclonal antibodies of the IgG type contain two identical antigen-binding "arms" and a constant fragment (Fc). Furthermore, the human IgG1 Fc domain can also play a role of fixing complement system. Recent findings have generated renewed interest in so-called "non-neutralizing" antibodies that are unable to directly inhibit free virus entry into target cells, but nonetheless, exhibit antiviral activity mediated by the Fc region of the antibody molecule. One class of antibody derivatives with the promise of enhanced potency for the treatment of disease is bispecific antibodies (BsAbs) that can bind to two distinct epitopes. Besides the dual-specific antigen binding fragment (Fab) parts, they contain an Fc portion and can thus be considered 'trifunctional' [26]. Antibodies with a dual specificity in their binding arms usually do not occur in nature and, therefore, had to be crafted with the help of recombinant DNA or cell-fusion technology. These antibody effector mechanisms include complement binding and viral lysis, phagocytosis of antibody-coated virions, and antibody-dependent cellular cytotoxicity [27,28]. Mark et al [10] reported a bispecific antibody to promote destruction of HIV-1 by neutrophils. The bispecific antibody incorporated the variable regions of the anti-gp41 antibody, F240, and the anti-CD89 antibody, 14A8. However, relatively little is known about the potential of complement-mediated destruction of HIV through bispecific antibody.

### The hypothesis

The hypothesis presented here investigates a new strategy using a bispecific antibody to target and amplify complement activation on HIV virions regardless of modulating complement inhibitor expression. The IgG-like bispecific antibody is constructed through incorporating the dsFv against gp120 and the dsFv against C3d, then linking to a complement-activator (human IgG1 Fc domain). The trifunctional bispecific antibody, (anti-gp120 × anti-C3d)-Fc, has two different antigen-specific binding sites, one for HIV gp120 and the other for the C3d. It is expected not only to target block HIV-gp120 and C3d on the surface of HIV, but also can enhance complement activation through the complement-activating Fc domain. Thus, (anti-gp120 × anti-C3d)-Fc can reduce the infectivity of HIV through blocking gp120 and C3d on the surface of HIV, since they can bind to their receptors and help virus to infect cells. Moreover, (anti-gp120 × anti-C3d)-Fc will inhibit the complement inhibitors (fH and CD59) binding to HIV that may enhance CoML. More importantly, this targeted complement activator is able to bind to sites of complement activation, so it is likely to improve their efficacy while reducing potentially serious side effects resulting from complement activation. Furthermore, the human IgG1 Fc domain can also play a role of repairing the complement system. Subsequently, the positive feedback loop generated by the complement cascade results in enhanced lysis of HIV and preventing infection of susceptible cells. Therefore, they represent a feasible approach against infection if they can be directed and armed to destroy HIV and infected cells.

## Testing of the hypothesis

After preparation of human (anti-gp120 × anti-C3d)-Fc fusion protein, biodistribution studies were performed to evaluate the biologic activity of (anti-gp120 × anti-C3d)-Fc *in vitro*. HIV-1 was cultivated in H9 cells and cell-free virus obtained from supernatants. Infection experiments were performed in 24-well plates in triplicate and virus opsonization was performed by incubating appropriate dilutions of HIV in culture medium with normal human serum (NHS) and purified (anti-gp120 × anti-C3d)-Fc proteins. A control group included viruses that were incubated with NHS only under the same conditions. Finally, neutralization tests were used to estimate the efficiency of (anti-gp120 × anti-C3d)-Fc enhanced lysis of HIV compared to controls.

## Implication of the hypothesis

A successful test of the hypothesis would demonstrate that (anti-gp120 × anti-C3d)-Fc can bind to HIV virions and can result in an amplification of the complement activation cascade. As a consequence of this action, HIV would likely be eliminated by CoML and further infection by HIV should be inhibited. Furthermore, (anti-gp120 × anti-C3d)-Fc bound to HIV virions is likely to reduce potential damage of host cells and tissues resulting from excess complement activation. Thus, it is meaningful to investigate the potential role of (anti-gp120 × anti-C3d)-Fc for the abrogation of HIV infection in humans, as this new finding would suggest another novel approach for HIV therapy.

## Competing interests

The authors declare that they have no competing interests.

## Authors' contributions

LLJ, YYX and HBS prepared the manuscript. CFZ, YW, SFQ, LGW, YWZ, WJL, YSS, FQ, ST, YSZ, YXH participated in developing the hypothesis and collaborated in writing and reviewing of the article. All authors read and approved the final manuscript.
